# CDNF and MANF in the brain dopamine system and their potential as treatment for Parkinson’s disease

**DOI:** 10.3389/fpsyt.2023.1188697

**Published:** 2023-07-24

**Authors:** Emmi Pakarinen, Päivi Lindholm

**Affiliations:** Institute of Biotechnology, Helsinki Institute of Life Science, University of Helsinki, Helsinki, Finland

**Keywords:** CDNF, MANF, dopamine, Parkinson’s disease, unfolded protein response

## Abstract

Parkinson’s disease (PD) is a progressive neurodegenerative disease characterized by gradual loss of midbrain dopamine neurons, leading to impaired motor function. Preclinical studies have indicated cerebral dopamine neurotrophic factor (CDNF) and mesencephalic astrocyte-derived neurotrophic factor (MANF) to be potential therapeutic molecules for the treatment of PD. CDNF was proven to be safe and well tolerated when tested in Phase I-II clinical trials in PD patients. Neuroprotective and neurorestorative effects of CDNF and MANF were demonstrated in animal models of PD, where they promoted the survival of dopamine neurons and improved motor function. However, biological roles of endogenous CDNF and MANF proteins in the midbrain dopamine system have been less clear. In addition to extracellular trophic activities, CDNF/MANF proteins function intracellularly in the endoplasmic reticulum (ER), where they modulate protein homeostasis and protect cells against ER stress by regulating the unfolded protein response (UPR). Here, our aim is to give an overview of the biology of endogenous CDNF and MANF in the brain dopamine system. We will discuss recent studies on CDNF and MANF knockout animal models, and effects of CDNF and MANF in preclinical models of PD. To elucidate possible roles of CDNF and MANF in human biology, we will review CDNF and MANF tissue expression patterns and regulation of CDNF/MANF levels in human diseases. Finally, we will discuss novel findings related to the molecular mechanism of CDNF and MANF action in ER stress, UPR, and inflammation, all of which are mechanisms potentially involved in the pathophysiology of PD.

## Introduction

PD is a progressive neurodegenerative movement disorder where dopamine neurons in the midbrain degenerate and are gradually lost. Aging is the main risk factor for PD, and incidence of PD is markedly increased in people over the age of 65 years (1). Characteristic motor symptoms of PD are resting tremor, slowness of movement, rigidity, and postural instability. The main neuronal population affected in the brain of PD patients is dopamine neurons located in the ventral midbrain, which regulate voluntary movement. Dopamine neurons have cell bodies in the substantia nigra (SN), where they project axons to the striatum, forming the nigrostriatal dopaminergic pathway. Characteristic brain pathology in the vast majority of PD patients is the presence of intraneuronal Lewy body inclusions, which contain aggregated α-synuclein (αSyn) protein (2). Clinical diagnosis of PD is based on motor symptoms, which manifest when degeneration of dopamine neurons is already severe. At the time of clinical diagnosis of PD, there is an estimated 30% reduction of dopamine neurons in the SN, and about 50%–60% of striatal dopamine-releasing axon terminals have been lost (3). Dopamine neurons are also present in the enteric nervous system (ENS), and their function can be affected in PD (4). Indeed, PD is not only a motor disease with patients suffering from various non-motor symptoms, including constipation, REM sleep behavior disorder, hyposmia, and depression etc., which can severely impair the quality of life and often appear years before the actual clinical symptoms (5). Despite extensive research efforts, no therapies are available for PD that would slow down or halt the progressive neurodegeneration.

Neurotrophic factors (NTFs) are small secretory proteins that support neuronal survival and promote formation and maintenance of neuronal contacts. They regulate proliferation, migration, differentiation, and maturation of neurons during development and synaptic plasticity in adults (6–8). Because of these beneficial properties, NTFs are studied as potential therapeutic molecules in PD. Glial cell line-derived neurotrophic factor (GDNF)-family ligands (GFLs), GDNF (9) and neurturin (NRTN) (10), are potent survival factors for midbrain dopamine neurons (7). GFLs function as covalently linked homodimers that bind to GDNF family receptor alpha (GFRα) co-receptors and activate transmembrane protein receptor tyrosine kinase RET on the plasma membrane, leading to activation of signaling cascades that promote neuronal survival and neurite outgrowth. However, when GDNF and NRTN were tested in Phase II clinical trials in PD patients, the outcomes of the trials were modest (11).

CDNF and MANF proteins form a unique family of trophic factors and function in many cell types. They have neurotrophic activity and show protective effects on midbrain dopamine neurons in animal models of PD *in vivo* (12–17). MANF (alternative name arginine-rich, mutated in early-stage tumors; ARMET) was originally isolated and purified from the culture medium of mesencephalic astrocyte-derived cell line based on its ability to specifically promote the survival of cultured midbrain dopamine neurons (18). CDNF is a vertebrate-specific paralog of MANF (12). In cells, CDNF and MANF localize in the endoplasmic reticulum (ER) (19, 20), where they regulate protein homeostasis and ER stress-induced unfolded protein response (UPR) signaling (21–25). As a potential therapeutic factor for dopamine neurons, CDNF was tested in Phase I-II clinical trials in 17 PD patients, where it was shown to be safe and well tolerated (26, 27).

## ER stress and UPR are possible pathogenic mechanisms in PD

The ER is an organelle important for protein synthesis, folding and post-translational modification, lipid synthesis, and calcium storage. Disturbances in the ER function caused by various physiological alterations and pathological conditions can lead to ER stress, a condition where unfolded and misfolded proteins accumulate in the ER (28). Dysregulations in protein homeostasis are associated with aging and can contribute to age-related neurodegenerative protein misfolding disorders, including PD (29).

**Figure 1:**
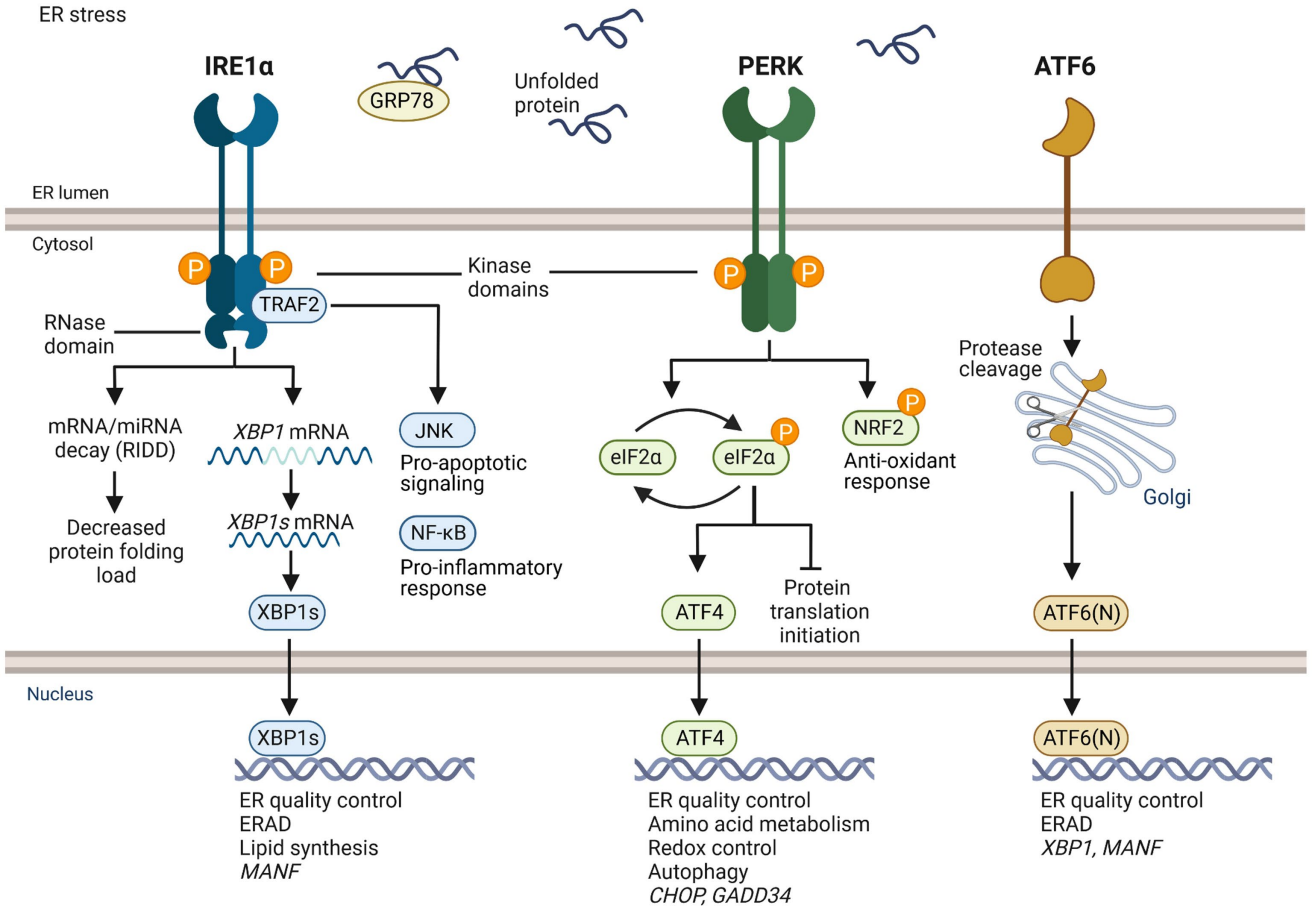
Overview of UPR signaling. IRE1α, PERK, and ATF6 are transmembrane receptors in the ER of mammalian cells that are activated upon ER stress. IRE1α and PERK have ER luminal domains and cytosolic kinase domains. IRE1α has also a cytosolic RNase domain. ATF6 has a luminal and a cytosolic domain, but no kinase activity. GRP78 is thought to be bound to the luminal domain of UPR receptors in homeostatic conditions. When unfolded proteins accumulate in the ER causing ER stress, GRP78 dissociates and binds unfolded client proteins, which allows activation of the UPR. Activated IRE1α forms homodimers leading to activation of cytosolic kinase domain, trans-autophosphorylation, and stimulation of cytosolic RNase domain. The activated RNase domain of IRE1α unconventionally splices *XBP1* mRNA to *XBP1s*, which is translated and functions as a transcription factor. XBP1s induces genes related to ER quality control, ER associated degradation (ERAD), and lipid synthesis. The RNase domain can degrade mRNAs and miRNAs through regulated IRE1α-dependent decay (RIDD). IRE1α adapter TRAF2 mediates c-Jun N-terminal kinase (JNK) activation and apoptotic signaling, and NF-κB activation and inflammatory response. Active PERK phosphorylates an α-subunit of eukaryotic initiation factor 2 (eIF2) leading to transient block of general translation initiation while transcription factor ATF4 is still translated. It induces genes of protein folding, redox control, amino acid metabolism and autophagy. Under unresolved ER stress, ATF4 induces pro-apoptotic transcriptional factor C/EBP homologous protein (CHOP) and growth arrest and DNA damage-inducible 34 (GADD34). GADD34 dephosphorylates elF2α reversing translational inhibition, which can aggravate ER stress and promote apoptosis. PERK also phosphorylates nuclear factor erythroid 2-related factor 2 (NRF2), a transcription factor that regulates antioxidant response. Activated ATF6 moves to the Golgi and is cleaved by endopeptidases, which releases ATF6(N) fragment. ATF6(N) induces expression of XBP1 mRNA and components of ERAD. MANF expression is induced by XBP1s and ATF6(N).

ER stress activates the unfolded protein response (UPR) signaling pathway that functions as an adaptive mechanism to restore ER protein homeostasis (Figure 1) (28, 30). Activated UPR leads to transient inhibition of general mRNA translation initiation, which decreases protein synthesis and protein folding load in the ER. UPR signaling also leads to increased expression of various chaperones that assist protein folding in the ER and increased degradation of misfolded proteins by ER-associated protein degradation (ERAD) in the cytosol. Although UPR is a homeostatic mechanism, in conditions of severe unresolved ER stress UPR signaling can become chronic, which leads to activation of UPR-mediated apoptotic pathways. Importantly, chronic UPR activation can lead to death of cells and neurons contributing to development of different pathologies, including neurodegenerative diseases (28).

Inositol-requiring enzyme 1 (IRE1; also known as ERN1), protein kinase R-like ER kinase (PERK; also known as EIF2AK3) and activating transcription factor 6 (ATF6) are ER transmembrane receptors in mammalian cells that monitor protein folding homeostasis in the ER and sense the accumulation of misfolded proteins via their luminal domains. IRE1 is the most conserved of the UPR sensors and was originally discovered in yeast (31). IRE1α contains cytosolic kinase and endoribonuclease (RNase) domains. When activated, IRE1α oligomerizes and *trans*-autophosphorylates leading to the activation of cytosolic RNase domain. The RNase domain of activated IRE1α unconventionally splices *XBP1* mRNA to *XBP1s* in the cytosol (32). *XBP1s* is translated to a transcription factor that induces expression of genes related to ER protein folding and quality control, ERAD, and lipid synthesis. In addition to *XBP1*, RNase of IRE1α can degrade other ER-targeted mRNAs and miRNAs through regulated IRE1α-dependent decay (RIDD) activity (33). Upon activation, PERK phosphorylates the α subunit of eukaryotic translation initiation factor 2 (eIF2α), which leads to transient arrest of translation initiation and decrease in the load of newly synthesized proteins in the ER (34). However, activating transcription factor *ATF4* mRNA and other mRNAs that have upstream 5′ open reading frames can still be translated (35). ATF4 regulates expression of genes related to protein quality control, amino acid metabolism, antioxidant response, and autophagy. ATF4 can induce expression of CHOP (36), which is an important transcription factor activating apoptosis related genes. ATF4 also induces expression of GADD34, which regulates protein phosphatase 1 and dephosphorylation of eIF2α (37). In unresolved ER stress and sustained UPR, ATF4 and CHOP contribute to increased protein synthesis, which further exacerbates protein folding stress and can lead to cell death. Differently from IRE1α and PERK, activated ATF6 moves to the Golgi, where it is specifically cleaved, releasing transcription factor ATF6(N) (38). ATF6(N) travels to the nucleus and induces expression of genes of protein folding and ERAD (35).

An ER chaperone, GRP78 (BiP) is important in regulating the activation of UPR signaling. Binding of GRP78 to the luminal domain of UPR sensors is thought to keep the sensors inactive (39). When unfolded proteins accumulate in the ER, GRP78 disassociates from the sensors to bind unfolded proteins, enabling dimerization and oligomerization of IRE1α and PERK. IRE1α and PERK may also directly bind unfolded proteins, leading to activation UPR signaling (40, 41).

**Figure 2:**
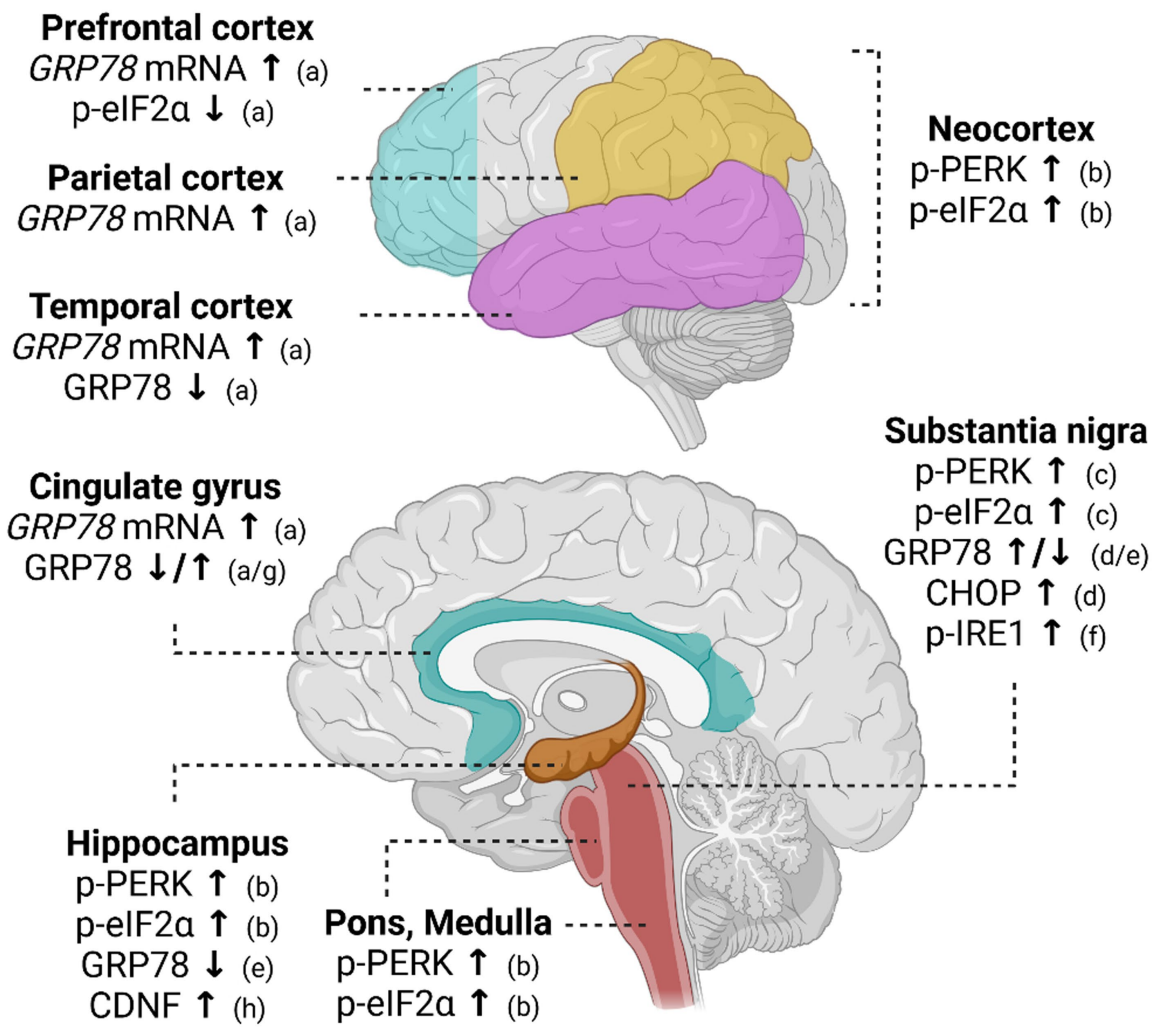
Altered UPR signaling in post-mortem brain of Parkinson’s disease patients. In the post-mortem PD brain, *GRP78* mRNA levels are increased in various cortical regions. Expression of GRP78 protein, in turn, has been shown to decrease in the hippocampus and temporal cortex of PD patients. In the substantia nigra and cingulate gyrus, GRP78 is shown to be either increased or decreased. Activation of PERK pathway measured as increased phosphorylation of PERK (p-PERK) and eIF2α (p-eIF2α) is implicated in the neocortex, hippocampus, pons, and medulla. Activation of both IRE1α and PERK pathways has been discovered in the substantia nigra. References indicated in the figure: a (46); b (43); c (42); d (44); e (47); f (48); g (45); h (59). **↑** indicates an increase in expression and **↓** a decrease in expression.

Studies using human post-mortem brain tissue indicate PD-associated changes in the regulation of UPR (Figure 2). Phosphorylation of PERK (p-PERK) and eIF2α (p-eIF2α) was demonstrated in the nigral dopamine neurons of PD patients using immunohistochemistry (42). Importantly, p-PERK was detected in neurons positive for αSyn, supporting the involvement of ER stress in PD pathology (42). In a post-mortem study of PD patients and cases of incidental Lewy body disease, activation of the PERK pathway was detected in connection to Lewy body pathology in several brain regions, including the midbrain, neocortex, hippocampus, pons, and medulla oblongata (43). In support of UPR activation in PD brain, increased levels of GRP78 and CHOP were detected in samples from the SN region of PD patients (44). In another study, increased levels of GRP78 were also demonstrated in the cingulate gyrus and parietal cortex of patients with dementia with Lewy bodies and Parkinson’s disease dementia relative to patients with Alzheimer disease, suggesting that UPR activation is more pronounced in cases with Lewy body pathology or when Lewy pathology is present with plaque pathologies (45). In the following study, decreased levels of GRP78 were reported in the temporal cortex and cingulate gyrus of PD patients. Although in contrast to the previous report, these results support the idea that impairments in the regulation of GRP78 expression may be related to PD pathophysiology (46). Decreased levels of GRP78 were also detected in the substantia nigra pars compacta (SNpc) and hippocampus of late-stage PD patients (47). Furthermore, detection of αSyn and phosphorylated IRE1α was reported in neuromelanin-containing nigral neurons of PD cases, reflecting the activation of the IRE1α pathway in PD pathology (48). Activated UPR is evidently related to brain pathology in PD, but its role in the onset and pathological progression of PD is completely unclear. Furthermore, it remains unknown how UPR is activated in PD. To the best of our knowledge, there are no published data on CDNF and MANF expression in association with the expression of UPR markers in the human PD brain.

## CDNF and MANF tissue expression

Tissue expression of endogenous CDNF and MANF in mammals has mainly been investigated in rodents, while studies reporting CDNF/MANF distribution in human tissues, particularly in the brain, are scant. When *CDNF* and *MANF* expression in adult human post-mortem brain tissue was studied by reverse transcription PCR (RT-PCR), *CDNF* (12) and *MANF* (49) transcripts were detected in all brain regions examined, including the caudate nucleus, putamen, and SN of the nigrostriatal dopamine pathway.

In the adult mouse brain, *Cdnf* mRNA and protein expression was detected in neurons of several brain areas (12). Immunohistochemical staining indicated CDNF expression in cortical neurons, hippocampus, striatum, and cerebellar Purkinje cells. CDNF immunosignal was also detected in the SN, although not co-localizing with tyrosine hydroxylase (TH) -positive dopamine neurons (12). MANF expression in the rodent brain was also mainly neuronal and detected in diverse brain regions, including the cerebral cortex, hippocampus, hypothalamus, thalamus, and cerebellum (49, 50). In the midbrain, immunohistochemical signal indicating MANF expression was detected in TH-positive dopamine neurons in the SNpc, but also in nigral TH-negative neurons, indicating that MANF expression is not restricted to dopamine neurons in the SN (50). MANF levels were generally higher in early postnatal stages of rat brain development and lower in adult rats, suggesting that MANF expression is developmentally regulated (50). A similar regulated pattern of MANF expression was also observed in the SN and caudate-putamen brain areas in the rat (50). MANF is also expressed in TH-positive dopamine neurons in the rodent ventral tegmental area, which is part of the mesolimbic pathway involved in reward-related behaviors (51). High expression of MANF was detected especially in brain neurons regulating energy homeostasis and endocrine functions (51).

In non-neuronal tissues, CDNF and MANF are ubiquitously expressed, but their levels differ depending on tissue and cell type. Tissue levels of CDNF are generally lower than those of MANF, except in skeletal muscle (51, 52). Especially high CDNF levels were also detected in the heart and brown adipose tissue (12, 51). MANF expression is particularly high in adult mouse tissues with metabolic and secretory functions, including the pancreas, liver, salivary gland, and testis (49, 51).

MANF and CDNF are present in blood circulation in humans and mice (51, 53–55), and circulating MANF levels were shown to decrease in human aging (56). In human blood cell fractions, MANF levels were high in platelets and lower in white blood cells (54). In the immune system, MANF expression was detected in plasma cells and macrophages in the human spleen, suggesting that MANF functions in plasma cell differentiation and immune system regulation (57).

## CDNF and MANF expression in human diseases

Changed levels of endogenous CDNF and MANF have been reported in numerous diseases (Table 1). Expression of endogenous MANF is increased especially in many inflammatory and metabolic diseases. Analyses have mostly focused on changes in protein levels in the serum or plasma, naturally due to availability of these samples. In the serum, MANF levels are increased in conditions such as ischemic stroke, drug-induced liver injury, hyperlipidemia, obesity, and types 1 and 2 diabetes (53, 61, 64, 66, 69, 73). In contrast, decreased levels of MANF in the serum have been reported in adult patients with growth hormone deficiency, major depressive disorder, nonalcoholic steatohepatitis, and polycystic ovary syndrome (56, 62, 68, 70). Expression of CDNF has been investigated in different disease conditions, but not as extensively as MANF. Findings indicate reduced expression of *CDNF* in the liver of patients with hepatocellular carcinoma and type 2 diabetes and in platelets of male stroke patients (58, 60).

**Table 1:** Human diseases reported with altered levels of endogenous CDNF and MANF.

Disease	Tissue	Measure	Change	References
CDNF				
Hepatocellular carcinoma	Liver	mRNA	**↓**	(58)
Parkinson’s disease	Hippocampus	Protein	**↑**	(59)
Parkinson’s disease	Blood	mRNA	**↑**	(54)
Stroke	Platelets	mRNA	**↓**	(60)
Type 2 diabetes	Liver	mRNA	**↓**	(58)
MANF				
Acute ischemic stroke	Serum	Protein	**↑**	(61)
Adult growth hormone deficiency	Serum	Protein	**↓**	(62)
Alzheimer’s disease	Cortex	Protein	**↑**	(63)
Drug-induced liver injury	Serum and liver	Protein	**↑**	(64)
Hepatitis B	Liver	mRNA, protein	**↑**	(65)
Hepatocellular carcinoma	Liver	mRNA	**↑**	(58)
Hyperlipidemia	Serum	Protein	**↑**	(66)
Inflammatory bowel disease	Colon	Protein	**↑**	(67)
Nonalcoholic steatohepatitis	Serum	Protein	**↓**	(56)
Major depressive disorder	Serum	Protein	**↓**	(68)
Obesity	Serum	Protein	**↑**	(69)
Parkinson’s disease	Serum	Protein	**↑**	(54)
Polycystic ovary syndrome	Serum	Protein	**↓**	(70)
Rheumatoid arthritis	White blood cells	mRNA	**↑**	(71)
Rheumatoid arthritis	Synovial membrane	Protein	**↑**	(72)
Systemic lupus erythematous	White blood cells	mRNA	**↑**	(71)
Transient ischemic attack	Serum	Protein	**↑**	(61)
Type 1 diabetes	Serum	Protein	**↑**	(53)
Type 2 diabetes	Serum	Protein	**↑**	(73)
Type 2 diabetes	Liver	mRNA	**↑**	(58)
Type 2 diabetes	Serum	Protein	**↑**	(66)
Type 2 diabetes	Plasma	Protein	**↓**	(74)

Diseases are listed in alphabetical order. **↑** indicates an increase in expression and **↓** a decrease in expression.

Interestingly, levels of CDNF and MANF are also altered in PD (Table 1). Analysis of post-mortem brain tissue indicates that expression of CDNF was increased in the hippocampus of PD patients (59). Hippocampal levels of CDNF were not associated with levels of total αSyn or phosphorylated αSyn – a modification implicated in PD pathology. In comparison, MANF levels remained unaltered in the hippocampal samples of PD patients (59). Instead, MANF levels were doubled in the serum of PD patients in comparison with the control group (54). Furthermore, circulating MANF levels correlated positively with the depression rating of PD patients. Analysis of whole blood did not show differences in *MANF* levels between patients and controls, whereas *CDNF* levels were slightly increased in PD patients (54). The source of MANF and CDNF in the blood is currently unclear, and whether their circulating levels could directly reflect changes in brain tissue or brain function remains to be investigated. Furthermore, it would be worthwhile to characterize possible changes in CDNF and MANF expression levels specifically in dopamine neurons and at different stages of PD progression.

Thus far, there are no known mutations in the *CDNF* and *MANF* genes associated with the risk for developing PD. In the *CDNF* gene, however, there is a single-nucleotide polymorphism (SNP) identified in early onset PD patients (75). This polymorphism was suggested to be associated with susceptibility for PD. Furthermore, another SNP in the *CDNF* gene was discovered to be a susceptibility locus in schizophrenia (76). Similar to PD, changes in the brain dopamine system play an important role in the pathogenesis of schizophrenia. Dysregulation of the dopamine system and more specifically diminished dopamine D1 receptor function in the prefrontal cortex have been associated with negative symptoms of schizophrenia patients (77). Interestingly, a SNP in the *CDNF* gene was associated with negative symptoms of schizophrenia patients in the study population (76).

There are two known individuals carrying autosomal recessive loss-of-function mutations in the *MANF* gene (78, 79). The identified individuals have protein-truncating variants in exon 1 of the *MANF* gene (78). These subjects presented with childhood onset syndromic diabetes with short stature, deafness, microcephaly, and developmental delay (78, 79). One of these subjects additionally has hypopituitarism, obesity, and partial alopecia (79). Sensorineural hearing loss was diagnosed in another patient already at the age of 11 months (80). Neither of the *MANF* variants that the patients carry has been detected in any of the tested population databases, indicating that homozygous variants are rare and result in phenotypes too severe to survive in humans (78).

## CDNF and MANF mouse models

The first CDNF knockout (KO) mouse model was reported in the year 2020 by Lindahl and co-authors (81). Conventional *Cdnf^−/−^* mice were viable and fertile with a normal appearance and normal blood parameters (81). The nigrostriatal dopamine system analyzed in aged mice showed no signs of degeneration. Levels of dopamine and its metabolites were similar between one-year-old *Cdnf^−/−^* and wildtype (WT) mice. The relative number of dopamine neurons in the SN was also unaltered in one-year-old *Cdnf^−/−^* mice, and still in 2-year-old *Cdnf^−/−^* mice. Furthermore, TH- and dopamine transporter (DAT)-positive fiber densities remained similar between *Cdnf^−/−^* and WT mice. Spontaneous locomotor activity was also not altered in *Cdnf^−/−^* mice, but when dopamine neurons lacking CDNF were induced to secrete high amounts of dopamine in response to amphetamine, their functionality was impaired. Amphetamine releases stored dopamine from vesicles, increasing cytosolic dopamine levels in dopamine neurons, and induces DAT-mediated dopamine efflux (82, 83). Amphetamine administration increased the activity of *Cdnf*^−/−^ mice compared with control mice, measured as distance traveled in the open field arena (81). This alteration in dopaminergic transmission manifested already at a young age but became more prominent in older *Cdnf^−/−^* mice. The effect seemed to be specific to amphetamine, as administration of a cocaine-mimicking compound did not cause a similar effect. The authors hypothesized that the mechanism would relate to DAT activity since amphetamine induces DAT reversal whereas cocaine blocks DAT (84). Therefore, dopamine release at the dorsolateral striatal dopamine terminals of *Cdnf^−/−^* mice was analyzed in more detail by fast-scan cyclic-voltammetry (81). Results showed that dopamine is released at similar levels and with similar kinetics in *Cdnf^−/−^* and control mice, but adult *Cdnf^−/−^* mice had slower dopamine reuptake. Incubation of striatal slices with amphetamine revealed that DAT reversed faster in *Cdnf^−/−^* mice, which is in line with observations made *in vivo*. Furthermore, more dopamine was released through DAT in amphetamine-stimulated striatal slices of *Cdnf^−/−^* mice.

While midbrain dopamine neurons maintain normal morphology, enteric neurons present various defects in *Cdnf^−/−^* mice. In general, enteric neurons are located in the submucosal and myenteric plexuses, which are interconnected layers in the gut. CDNF is expressed in neuronal subsets of both plexuses along the gut (85). Lack of CDNF caused neuronal hypoplasia in the submucosal plexus but not in the myenteric plexus (81). Lower neuronal density in *Cdnf^−/−^* mice was due to increased non-apoptotic neurodegeneration following enhanced autophagy (81). In the ileum, which has the highest dopamine neuron density, DAT transcripts were reduced in *Cdnf^−/−^* mice already at 1 week of age, suggesting impaired development of dopamine neurons (85). Together with general neuronal hypoplasia, the density of dopamine neurons was reduced in *Cdnf^−/−^* mice already at the age of 1.5 months. Furthermore, an age-dependent decline in the number of dopamine neurons was exacerbated in mice lacking CDNF. However, the defect was not specific to dopamine neurons, also impacting nNOS-, GABA- and CGRP-positive neurons in the submucosal plexus (85). At the functional level, loss of CDNF and consequent loss of dopamine neurons in the submucosal plexus controlling peristaltic activity caused delayed gastric emptying, slower colonic motility, and a longer transit time in 11-month-old mice (85). Late functional deficits observed in the gut of *Cdnf^−/−^* mice reflect the importance of CDNF in maintenance of the ENS.

In addition to *in vivo* observations in embryonic and adult CDNF-deficient mice, *in vitro* experiments using exogenous recombinant CDNF protein showed a difference in the survival-promoting effect of CDNF between midbrain and enteric dopamine neurons. CDNF did not promote the survival of dopamine neurons isolated from embryonic mouse midbrain floor (20). Instead, CDNF promoted specifically the development/survival of dopamine neurons isolated from mouse enteric neural crest-derived cells (85).

Conventional *Manf^−/−^* mice in an outbred ICR strain develop diabetes during the first months of life and die young (25). In contrast, *Manf^−/−^* mice in the inbred C57BL/6 strain are perinatal lethal (86–88). The diabetic phenotype of *Manf^−/−^* mice in the ICR strain is caused by the decreased proliferation and loss of pancreatic insulin-producing beta cells due to persistent ER stress (25, 51). Postnatal *Manf^−/−^* mice have normal glucose levels but start to lose beta cells soon after birth followed by hyperglycemia (25). *Manf^−/−^* mice in the ICR strain or in the mixed C57BL/6 J:129SV strain present with a visible growth defect related to disturbances in bone growth and impaired function of the pituitary gland (51, 87). Furthermore, *Manf^−/−^* mice are characterized as having hearing loss (89). These phenotypes – diabetes, growth defect, and hearing loss – are noticeably similar to symptoms found in human subjects with MANF loss-of-function mutations (25, 51, 78, 80, 89).

Investigation of the role of MANF removal in the dopamine system was performed with *Manf^fl/fl^::Nestin^Cre/+^* mice, devoid of loss of pancreatic MANF and consequent hyperglycemia. Nestin Cre-mediated removal of MANF expression applies to neuronal and glial cell precursors. Results showed that MANF deficiency did not affect the relative number of dopamine neurons in the SN, TH- or DAT-positive fiber density in the striatum, or dopamine and its metabolite levels in aged *Manf^fl/fl^::Nestin^Cre/+^* mice (90). MANF ablation also did not result in changes in expression of TH or DAT in the midbrain. Furthermore, the response to amphetamine or motor behavior was similar between *Manf^fl/fl^::Nestin^Cre/+^* and control mice measured both at young and old age (90).

As CDNF and MANF are structurally highly similar proteins, the question of possible redundancy was raised. To investigate possible functional compensation between CDNF and MANF, mice lacking both proteins were produced (52). The phenotype of *Cdnf^−/−^::Manf^−/−^* mice is dominated by diabetes and a growth defect, resembling the phenotype of *Manf^−/−^* mice. The early onset of diabetes prevented analysis of dopamine neuron maintenance in *Cdnf^−/−^::Manf^−/−^* mice; however, the *Cdnf^−/−^::Manf^−/−^* mouse brain was investigated during development. At embryonic day 13.5, at the time when CDNF and MANF are already expressed (12, 49), the *Th* levels in the brain were shown to be similar between *Cdnf^−/−^*::*Manf^−/−^* and control mice despite the already increased UPR activation in the brain of *Cdnf^−/−^*::*Manf^−/−^* mice (52). At postnatal day 1, TH protein levels were not changed in the *Cdnf^−/−^*::*Manf^−/−^* mouse brain, remaining unaltered even at 6 weeks of age (52). However, a more extended analysis of the maturation of the midbrain dopamine system would be important since it has been shown that MANF is essential for neuronal migration in the cortex during development (91). The distribution of cortical layer markers is altered in the neocortex of *Manf^−/−^* mice, and neuronal migration is delayed in *Manf^−/−^* embryos, although the number of cortical neurons is not reduced and MANF is dispensable for neurogenesis (91). Whether similar developmental delays take place during maturation of dopamine neurons remains to be investigated. To examine the effect of CDNF and MANF ablation on the survival of dopamine neurons in the long term, *Manf^fl/fl^::Nestin^Cre/+^* mice lacking CDNF were bred (52). The simultaneous deficiency of neuronal MANF and CDNF had no effect on dopamine neuron number in the SN or TH-positive fiber density in the striatum of one-year-old mice. Based on these findings, endogenous CDNF and MANF are not significant survival factors for nigrostriatal dopamine neurons in mice, but CDNF regulates their function.

One of the most significant differences in the dopamine system between CDNF and MANF KO mice is the presence of increased UPR activation in MANF KO mice relative to WT mice. Loss of MANF was reported to induce expression of *sXbp1* and *Grp78* mRNA in the striatum and in the SN of 5-week-old conventional *Manf^−/−^* mice (90). In addition, UPR-related genes *Atf4*, *Erdj4*, *Pdia6*, and *Grp94* were also upregulated in the striatum of *Manf^−/−^* mice (52). Similar to *Manf^−/−^* mice, adult *Manf^fl/fl^::Nestin^Cre/+^* mice had increased expression of *sXbp1* and *Grp78* in the nigrostriatal pathway (90). Thus, the increase in the activation of the IRE1α pathway and *Grp78* expression are persistent through development and aging and in fact, throughout different brain regions of MANF KO models. Aged *Manf^fl/fl^::Nestin^Cre/+^* mice also had modestly increased mRNA levels of *Atf4* and *Atf6* in the SN, reflecting activation of other PERK and ATF6 pathways (52). In contrast, lack of CDNF did not affect the UPR signaling in the brain of *Cdnf^−/−^* mice (52, 81). Furthermore, lack of CDNF did not aggravate impaired proteostasis caused by loss of MANF alone (52). Instead, CDNF ablation resulted in increased UPR activation in the skeletal muscle and lack of MANF aggravated this increase (52). Thus, loss CDNF and MANF affect UPR status *in vivo*, but in a tissue-specific manner.

Although MANF deficiency alone does not induce neuronal loss (90, 92), MANF deficiency increases neuronal vulnerability, which was shown to manifest when *Manf^fl/fl^::Nestin^Cre/+^* mice were subjected to such stressors as ethanol, tunicamycin, or blockage of blood flow in the stroke model (92, 93). When neuronal apoptosis was induced in one-week-old *Manf^fl/fl^::Nestin^Cre/+^* mice by ethanol or tunicamycin, these exposures resulted in a higher increase in apoptosis and exacerbated ER stress in *Manf^fl/fl^::Nestin^Cre/+^* mice compared with control mice (92). Inhibiting ER stress by 4-phenylbutyric acid could abrogate increased neuronal apoptosis in *Manf^fl/fl^::Nestin^Cre/+^* mice subjected to the stressors. Thus, it was suggested that *Manf^fl/fl^::Nestin^Cre/+^* mice have a lowered buffering capacity for induced neuronal degeneration. In line with these observations, cortical neurons isolated from *Manf^−/−^* mice were shown to be more vulnerable to thapsigargin-induced ER stress in culture than neurons extracted from WT mice (90). Thus, it is plausible that dopamine neurons lacking MANF are also highly vulnerable to ER stress. Induction of ischemic infarct by arterial occlusion caused a larger infarction volume in *Manf^fl/fl^::Nestin^Cre/+^* mice than in control mice (93). When *Cdnf^−/−^* mice were subjected to hemorrhagic insult by collagen injection, the lesion volume was significantly larger than in control mice (94). Thus, loss of endogenous CDNF and MANF increases vulnerability to cerebral insults.

## CDNF, MANF, and other model organisms

A single homologous gene to vertebrate CDNF/MANF is found in invertebrates. The invertebrate homolog of CDNF/MANF, first discovered in the fruit fly, shows higher sequence similarity to MANF than CDNF (95). During development DmManf expression localizes specifically to glial cells, while adult flies express DmManf in both glia and neurons (95, 96). *DmManf* null mutants were reported to be late embryonic lethal with severe cuticular defects (95). *DmManf* mutants appeared to have a massive loss of dopamine neurites and low levels of dopamine, although, interestingly, neuronal somas persisted. In fruit flies, dopamine is required for cuticle formation, including cuticle pigmentation and sclerotization (97). Therefore, extremely low levels of dopamine might at least partially explain disorganization of cuticle layers in *DmManf* mutant flies. In contrast to *DmManf* mutant flies, removal of DmManf from neurons or glia did not affect morphology of dopamine neurons in the brain, indicating unaltered neuronal differentiation during development (96). Thus, DmManf was concluded to not be required in a cell-autonomous fashion for the survival of dopamine neurons in *Drosophila*. Furthermore, overexpression of DmManf in neurons or glia had no impact on the dopamine system of the fly (96).

MANF ortholog in *Caenorhabditis elegans* nematodes, manf-1, has a wide expression pattern, including the intestine and central nervous system (CNS) (98). Nematodes with mutated *manf-1* were reported to have no clear morphological defects but had a slower growth rate (99). Regarding the dopamine system, these *manf-1* mutant nematodes had a normal number of dopamine neuron somas and neurites in the first days of adulthood. However, from day 3 onwards, *manf-1* mutants started to lose dopamine neurons, accruing a loss of almost 30% by day 9 (99). In agreements with these results, Hartman and others (100) showed that their *manf-1* mutants had normal development in dopamine neurons, regular locomotion, and even similar sensitivity to the 6-OHDA toxin as controls in early adulthood. However, they observed that tunicamycin caused modulation of the innate immune system in *manf-1* mutants, making them resistant to the growth arrest observed in tunicamycin-treated WT nematodes. An increase in the expression of hsp-4 (an ortholog of human GRP78) was documented in the *manf-1* mutant nematodes (98, 99) and upregulation of UPR genes in *DmManf* mutant fruit flies (101), in line with observations of increased UPR activation in response to loss of MANF in mice.

Expression of both CDNF and MANF is present in zebrafish. In the zebrafish brain, MANF is expressed in neurons (102). Knocking down MANF expression by antisense splice-blocking morpholino oligonucleotides produced *manf* mutants with a rather normal appearance and normal swimming behavior (102). However, a detailed analysis of the *manf* mutant zebrafish revealed reduced dopamine levels and decreased expression of *th1* and *th2* transcripts, both of the duplicated *th* genes in zebrafish. Moreover, *manf* mutant fish had region-specific alterations in the TH1 and TH2 cell populations, now in the prethalamus and periventricular nucleus of the posterior tuberculum. The decrease in TH1-positive cell number and reduced dopamine levels could be rescued by manf mRNA injections to the embryos. Overexpression of manf in WT fish embryos, by contrast, had no effect on dopamine neuron development.

Zebrafish express CDNF predominantly in the eye and the brain, which differs from the expression pattern seen in mice. The physiological function of CDNF in zebrafish was addressed using CRISPR-Cas9-mediated removal of CDNF expression. Zebrafish lacking CDNF were discovered to have elevated levels of *th2* and normal *th1* levels in the brain (103). Furthermore, they had normal dopamine levels but a misbalance in dopamine neuron clusters; more TH2-positive neurons were present in the caudal hypothalamus and less TH1-positive neurons in the prethalamus. However, dopamine neurons were not the only neuron population affected by loss of CDNF, as both histamine and GABA neurons were reduced in the brain. Thus, lack of CDNF affects several neurotransmitter circuits in zebrafish. Furthermore, *cdnf* mutant zebrafish had increased swimming speed and altered shoaling pattern, indicating reduced sociability.

## CDNF, MANF, and *in vivo* PD models

Toxin-based animal models of PD are extensively used in preclinical studies to investigate possible treatments for the disease. Neurotoxins are used as tools to induce degeneration of the nigrostriatal pathway to mimic the dopamine neuron loss present in patients with PD. The widely used method is a unilateral lesion model caused by intrastriatal injections of 6-hydroxydopamine (6-OHDA), which destroys dopamine nerve terminals in the striatum and cell somas in the SN. Lesion-induced deficits are normally evaluated by drug-induced rotational behavior and quantification of the survival of dopamine neurons and their striatal fiber integrity. Compared with intrastriatal injections, 6-OHDA injections to the medial forebrain bundle (MFB) cause a more severe loss of dopamine neurons in the SN (104), modeling a late-stage PD. Another commonly used neurotoxin, 1-methyl-4-phenyl-1,2,3,4-tetrahydropyridine (MPTP), can be administered systemically and outcome measures include behavioral assessment in addition to the analysis of the dopamine neuron survival. Moreover, exposure to rotenone, an inhibitor of complex I, also causes neuropathological features of PD in rats (105). Instead of toxin, αSyn preformed fibril (PFF)-induced PD model involves injection of PFFs into the brain, which leads to formation of Lewy body-like aggregates (106). This model has become a valuable tool to examine the effect of potential therapeutic compounds on αSyn pathology.

At the time of its discovery, CDNF was tested in the rat 6-OHDA model of PD (Table 2). When CDNF protein was administered before the 6-OHDA-induced lesion, it could protect dopamine neurons from degeneration. The amphetamine-induced rotations were decreased and TH-positive fiber density in the striatum and the amount of dopamine neurons were higher in the SN than in vehicle-treated rats (12). Intrastriatal administration of CDNF after 6-OHDA lesion induction could also improve rotational asymmetry and survival of dopamine neurons in the SN. Thus, CDNF exhibited both protective and restorative activity for dopamine neurons. Two-week chronic delivery of CDNF also demonstrated similar restorative activity in the 6-OHDA model of PD by reducing amphetamine-induced rotational asymmetry and improving TH-positive cell survival (14). In a later study, a smaller dose of CDNF provided only modest protection of dopamine neurons (107). In the abovementioned studies, CDNF has mainly been delivered to the striatum. From the striatum, injected CDNF has been shown to diffuse well, reach the SN, and localize into dopamine neurons (14, 113). The half-life of CDNF in brain tissue is 5.5 h when injected intrastriatally (113).

**Table 2:** Summary of studies examining the effect of CDNF in the rodent models of Parkinson’s disease.

Disease model	Delivery method of CDNF	Time of CDNF delivery	Outcomes of CDNF therapy	References
6-OHDA to the striatum	Single protein injection to the striatum	Prior to lesion	Protection of TH+ neurons in the SN, TH+ fibers in the striatum, reduced rotations	(12)
		Post lesion	Improved survival of TH+ neurons in the SN, reduced rotations	
	Chronic protein delivery to the striatum	Post lesion	Reduced rotations, improved survival of TH+ neurons in the SN and TH+ fibers in the striatum	(14)
	Single protein injection to the striatum	Post lesion	Modest reduction in rotational behavior, no significant improvement in the survival of TH+ neurons or TH+ fiber density	(107)
	Gene delivery via AAV2 vector to the striatum	Prior to lesion	Reduced rotations, positive effect on survival of TH+ neurons in the SN	(16)
	Gene delivery via AAV2 vector to the striatum	Post lesion	Reduced rotations, improved survival of TH+ neurons in the SN and TH+ fibers in the striatum, improved DAT activity	(17)
	Gene delivery via AAV8 vector to the striatum	Post lesion (2 or 5 weeks)	Reduced rotations, improved TH+ fiber density and improved survival of TH+ neurons	(108)
	Gene delivery via lentivirus to the striatum	Time of the lesion	No significant improvement in the survival of TH+ neurons in the SN or striatal TH+ fibers, nor rotations	(109)
	Gene delivery via lentivirus to the SN	Time of the lesion	Improved survival of striatal TH+ fibers, reduced rotations	
	Delivery of CDNF-expressing mesenchymal stem cells to the striatum	Post lesion	Reduced rotations, improved survival of the TH+ neurons in the SN and density of TH+ fibers in the striatum	(110)
6-OHDA to the medial forebrain bundle	Single protein injection to the cerebral ventricle	Post lesion	No significant improvement in survival of TH+ neurons or rotational behavior	(111)
	Single protein injection to the SN	Post lesion	Reduced rotations, no significant improvement in survival of TH+ neurons or TH+ fibers	(112)
MPTP	Single protein injection to the striatum bilaterally	Prior to lesion	Protection of nigral TH+ neurons and striatal TH+ fibers, improved motor behavior	(15)
		Post lesion		

Reduced amphetamine- or apomorphine-induced rotations refer to a reduced lesion severity. AAV, adeno-associated virus; SN, substantia nigra; TH, tyrosine hydroxylase; DAT, dopamine transporter.

Several studies have investigated the effect of CDNF overexpression via virus vector-mediated delivery in the 6-OHDA model (Table 2). In general, enhancement of intracellular CDNF expression has been shown to be an efficient way to protect dopamine neurons in PD models. Adeno-associated virus 2 (AAV2)-mediated overexpression of CDNF in the striatum produced different levels of neuroprotection depending on the time of CDNF induction in relation to the 6-OHDA lesion. When CDNF was expressed before the 6-OHDA lesion, it had only a modest protective effect (16), but when delivered after 6-OHDA, CDNF was able to reduce amphetamine-induced rotations, improve the survival of the dopamine neurons in the SN, and even improve DAT activity (17). In another study, CDNF expression was induced via lentivirus vector in either the striatum or the SN. CDNF improved rotational asymmetry and dopamine fiber density only when it was expressed in the striatum (109). In the study of Wang et al. (108), AAV8-CDNF reduced amphetamine-induced rotational behavior and improved survival of TH-positive neurons more efficiently when administered 2 weeks after 6-OHDA than 5 weeks after lesion induction, reflecting mild and severe lesions, respectively. CDNF-expressing mesenchymal stem cells injected to the striatum was also shown to improve motor behavior and neuronal survival (110). In the mouse MPTP model of PD, CDNF—applied either before or after toxin—improved motor behavior and survival of nigral dopamine neurons (15).

Therapeutic efficacy of CDNF may depend on several factors such as the severity of PD model and the route of administration. After injections of 6-OHDA into the MFB, CDNF has not achieved neuronal restoration in rodent PD models (111, 112). CDNF could improve only apomorphine-induced rotational asymmetry (112). It is plausible that this model of late-stage PD is too severe for CDNF to rescue dopamine neurons. These data suggest that CDNF therapy would be more suitable for early-stage PD patients. While in most preclinical studies CDNF is delivered intrastriatally, these two studies used different routes for the delivery: the intracerebral ventricle (111) and the SN (112). In a naïve rat brain, CDNF injected to the SN is not transported to the striatum, indicating that anterograde transport of CDNF is not active, in contrast to the observed active retrograde CDNF transport (114). However, injected CDNF localizes into TH-positive neurons in the SN, enabling intracellular protective activity (114).

Interestingly, a recent study indicated that CDNF can bind αSyn *in vitro*, and CDNF injected into brain tissue was shown to interact with αSyn using proximity ligation assay (115). Furthermore, CDNF was shown to decrease αSyn internalization into primary hippocampal neurons (115). Neuroprotective effects of CDNF were also demonstrated against toxicity of αSyn oligomers in primary cultures of mesencephalic neurons and in dopamine neurons differentiated from the Neuro 2a cell line (116). Based on these findings, CDNF was tested in the PFF-induced rodent model of PD. Despite no loss of TH-positive neurons or degeneration of TH-positive fibers, CDNF improved paw use in the cylinder test, measuring asymmetry of paw use (115).

Many studies have reported of significantly improved outcomes when CDNF protein is administered with another protein or with another therapeutic strategy. For instance, CDNF treatment has been tested in combination with GDNF in the rat 6-OHDA model of PD (107). Co-administration of CDNF and GDNF reduced amphetamine-induced ipsilateral turns and protected TH-positive neurons in the SN more efficiently than proteins alone. CDNF overexpression has also been boosted with a simultaneous gene delivery of aromatic L-amino acid decarboxylase (AADC), which is an enzyme involved in dopamine synthesis and has been shown to alleviate symptoms of PD patients in clinical trials (117). Overexpression of AADC on top of CDNF increased striatal dopamine levels more efficiently than gene delivery of CDNF alone (108). Enhancing the effect of CDNF gene therapy by simultaneous MANF delivery promoted survival of TH-positive neurons in the SN and fibers in the striatum in addition to reduced rotational turns, suggesting a synergistic effect of CDNF and MANF on neurorestoration (109). An alternative strategy was presented in a study where CDNF injections were combined with a deep brain stimulation of the subthalamic nucleus (STN) in a rat MFB 6-OHDA model (112). Intranigral delivery of CDNF improved efficacy of subthalamic stimulation through implanted electrodes in a cylinder test and in the survival of TH-positive fibers. Delivery of CDNF was also combined with a STN lesion, modeling chronic stimulation of the nucleus, and results showed amelioration of motor deficits (112). More recently, CDNF was administered with ventral mesencephalic (VM) grafted tissue to rats with 6-OHDA injections to the MFB. When CDNF was co-administered with VM grafted tissue, it improved the outcome of cell transplantation (111).

Given that CDNF provided positive effects in preclinical studies of PD in rodents, the efficacy of CDNF was also tested in nonhuman primates with a parkinsonian model to validate its translational potential. Although CDNF did not increase the survival of TH-positive neurons in 6-OHDA-lesioned marmoset monkeys, there was an increase in DAT binding activity in 6-OHDA-lesioned animals treated with CDNF (118).

Neuroprotective activity of MANF has been tested in different PD models through protein and gene delivery, as summarized in Table 3. A single injection of MANF protein to the striatum reduced amphetamine-induced rotations and improved the survival of TH-positive neurons in the SN when applied before 6-OHDA in unilaterally 6-OHDA-lesioned rats (13). Furthermore, MANF delivery after 6-OHDA improved amphetamine-induced turning behavior in two independent studies (13, 119). Chronic delivery of MANF protein for 2 weeks, by contrast, did not alleviate rotational behavior or the survival of dopamine neurons compared with vehicle-treated animals (14). In the same study, chronic delivery of GDNF also did not promote neuroprotection (14). Inspection of intrastriatally injected iodinated MANF showed transport to the cortex but not to the SN, indicating effective retrograde and inefficient anterograde transport (14). While lentivirus vector-mediated MANF overexpression in the striatum did not promote dopamine neuron function or survival in 6-OHDA model (109), intrastriatal AAV9-mediated gene delivery of MANF, in turn, showed a significant improvement in 6-OHDA-treated rats (120). Hao and others (120) reported an increase in the survival of midbrain dopamine neurons and an increase in striatal dopamine levels in addition to reduced amphetamine-induced rotations. Similarly, AAV8-vector-mediated MANF expression in the striatum resulted in improved motor behavior and survival of TH-positive neurons in the SN in a 6-OHDA rat model of PD (121). Improved motor behavior, increased survival of TH-positive cells in the SN, and increased striatal dopamine levels were also observed in the mouse MPTP model of PD in response to MANF delivery to the striatum (122). In the rotenone model of PD, MANF gene transfer resulted in improved motor behavior and enhanced survival of TH-positive neurons in the SN (123).

**Table 3:** Summary of preclinical studies of MANF in rodent Parkinson’s disease models.

Disease model	Delivery method of MANF	Time of MANF delivery	Outcomes of MANF therapy	References
6-OHDA to the striatum	Single protein injection to the striatum	Prior to lesion	Reduced rotations, improved survival of TH+ neurons in the SN	(13)
		Post lesion	Reduced rotations, no significant improvement in the survival of TH+ cells in the SN	
	Single protein injection to the striatum	Post lesion	Reduced rotations, improved survival of TH+ neurons in the caudal part of SN	(119)
	Chronic protein delivery to the striatum	Post lesion	No significant improvement in rotations, TH+ cell survival or TH+ fiber density	(14)
	Gene delivery via AAV9 vector to the striatum	Post lesion	Promoted survival of TH+ neurons in the SN, improved TH+ fiber density, increased dopamine levels, reduced rotations	(120)
	Gene delivery via lentivirus vector to the striatum	Time of the lesion	No significant improvement in the survival of TH+ cells in the SN or striatal TH+ fibers, or in rotations	(109)
	Gene delivery via lentivirus vector to the SN	Time of the lesion	Improved survival of TH+ cells in the SN, no improvement in rotational behavior	
	Gene delivery via AAV8 vector to the SN	Prior to lesion	Improved motor behavior, improved survival of TH+ neurons in the SN, reduced activation of microglia	(121)
MPTP	Single protein injection to the striatum bilaterally	Time of the lesion	Improved motor behavior, increase in striatal dopamine and its metabolites, protection of TH+ cells in the SN	(122)
Rotenone	Gene delivery via AAV8 vector to the SN	Prior to lesion	Improved motor behavior, improved survival of TH+ cells in the SN	(123)

AAV, adeno-associated virus; SN, substantia nigra; TH, tyrosine hydroxylase.

Regarding the mechanism of action, it is important to note that increasing CDNF and MANF levels in a naïve rodent brain by protein injections has no or very modest effect on the dopamine system. Administration of CDNF or MANF does not affect TH-positive cell number in the SN, TH-positive fiber density in the striatum, or TH activity in the intact rat brain (15, 107, 124). In addition, CDNF injection does not alter TH or DAT expression or amphetamine-induced locomotor activity in naïve mice or rats (15, 107, 124). One finding indicates that MANF, but not CDNF, increases potassium- and amphetamine-evoked dopamine release and dopamine turnover, reflecting increased dopamine metabolism (124). Overall, these findings emphasize that the mechanism of action of CDNF and MANF warrants investigation in stressed cells.

## Molecular structure of CDNF/MANF proteins

**Figure 3:**
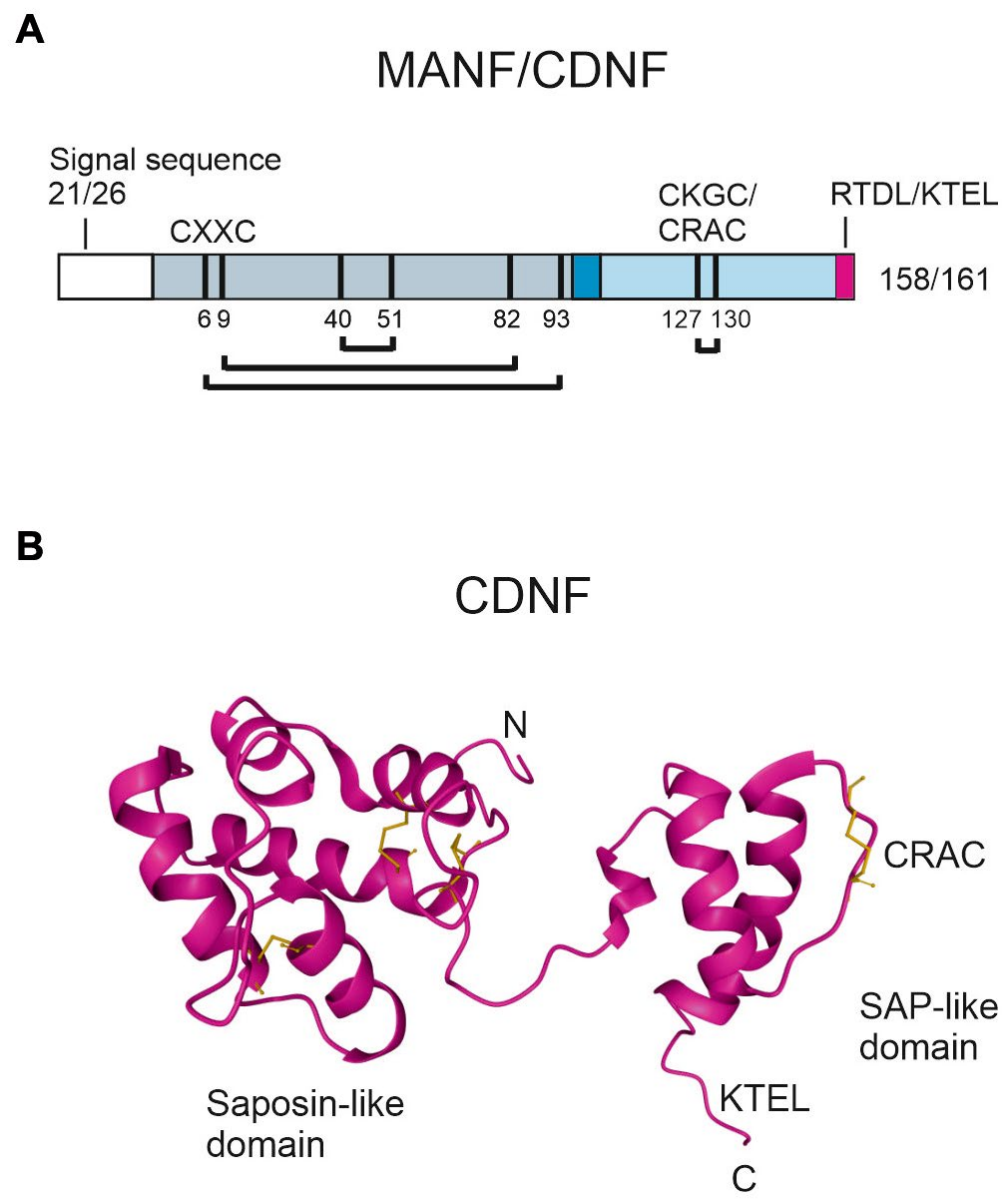
Structure of human MANF and CDNF proteins. **(A)** Schematic presentation of the primary structure of MANF/CDNF. After predicted cleavage of the signal peptide directing newly synthetized MANF/CDNF to the ER, mature MANF/CDNF consists of 158/161 amino acid residues, respectively. N-terminal saposin-like domain of mature protein is indicated in gray, linker region connecting the two domains in dark blue, and C-terminal SAP-like domain in light blue. A KDEL-like ER retention signal (pink) is located at the C-terminus. Eight conserved cysteines are indicated as black bars and numbered according to MANF. The cysteines can form four disulfide bridges, three in the N-terminal domain and one in the C-terminal domain (connecting lines). The C-terminal cysteine bridge is in CXXC motif (CKGC/CRAC in MANF/CDNF, respectively), which is crucial for MANF activity. **(B)** NMR solution structure of CDNF (PDB ID: 4BIT) (116). Disulfide bridges (in yellow) connect α-helices in the N-terminal domain, stabilizing the saposin-like fold. Characteristic for SAP-like proteins is the helix–loop–helix structure, which is a putative DNA binding motif.

CDNF/MANF family members are small, soluble proteins with a molecular weight of about 18 kDa that consist of two domains and a flexible linker region, allowing free movement of the domains (116, 125, 126). A characteristic feature in the primary structure of CDNF/MANF proteins is eight conserved cysteine residues, which form four cysteine bridges that are important for protein folding and biological activity (Figure 3A). The three-dimensional structure of human CDNF and MANF was resolved using x-ray crystallography and nuclear magnetic resonance (NMR) spectroscopy (116, 125–127). The amino (N)-terminal domain of CDNF/MANF contains three disulfide bridges and is conformationally similar to saposin-like proteins, which commonly interact with lipids and membranes (125, 126, 128) (Figure 3B). In accord with this, MANF was shown to interact with sulfoglycolipid 3-*O*-sulfogalactosylceramide (sulfatide), which is present in the outer leaflet of the plasma membrane of various cell types (98). In the nervous system, sulfatide is abundant in the myelin sheath of oligodendrocytes and Schwann cells and it is also present in neurons and astrocytes (129). Bai and others (98) suggested that sulfatide is associated with MANF during its secretion, and sulfatide binding facilitates MANF uptake to cells. MANF together with sulfatide alleviated ER stress of manf-1 null mutants in *C. elegans* and protected mammalian cell lines under ER stress and hypoxia-glucose deprivation. Unlike MANF, CDNF does not bind sulfatide (98), and lipid partners of CDNF have not been described to date. MANF was also reported to interact with lipid kinase phosphatidylinositol 5-phosphate 4-kinase type-2 beta (PIP4K2b), which phosphorylates phosphatidylinositol-5-phosphate to phosphatidylinositol-4,5-bisphosphate in the ER (130), thus connecting MANF to the biology of phosphoinositide lipids. MANF interaction with PIP4K2b was suggested to enhance PIP4K2b activity and regulate insulin signaling in the hypothalamus (130).

The carboxy (C)-terminal domain of CDNF/MANF proteins has a helix–loop–helix arrangement and is structurally similar to members of SAF-A/B, Acinus, and PIAS protein superfamily (SAP), which are putative DNA binding proteins (131) (Figure 3B). The SAP-like domain of CDNF/MANF is important for the neuroprotective activity. Recombinant C-terminal MANF protein lacking the N-terminal domain, which was microinjected to mouse superior cervical ganglion (SCG) sympathetic neurons, promoted neuron survival against etoposide-induced apoptosis *in vitro* (126). An important functional motif in the SAP-domain of CDNF/MANF is a cysteine-X-X-cysteine (CXXC) sequence that can be found in the catalytic center of thiol-disulfide oxidoreductases (132). Microinjected, intracellular MANF protein with a mutated CXXC motif CKGS in the SAP domain was inactive against etoposide- or thapsigargin-induced apoptosis in SCG or dorsal root ganglion (DRG) sensory neurons *in vitro* as well as *in vivo* in a rat model of stroke, where mutated MANF CKGS was injected into brain parenchyma (133). Another potential CXXC motif is located in the N-terminal domain of CDNF/MANF (Figure 3A). However, studies did not show oxidoreductase activity in MANF (133–135).

ER resident proteins have a characteristic lysine-aspartic acid-glutamic acid-leucine (KDEL) sequence that binds to KDEL receptor (KDELR), which functions in retrieving proteins from the Golgi back to the ER, preventing their secretion and maintaining ER homeostasis. KDEL-like sequence is located at the very end of the C-terminal domain of CDNF/MANF proteins (134, 136, 137) (Figures 3A,B). Deletion of the KDEL-like sequence, i.e., KTEL in CDNF and RTDL in MANF, respectively, increased CDNF and MANF secretion (136, 138, 139). Interestingly, it has been suggested that KDELR can translocate to the cell surface particularly during ER stress (136). In the plasma membrane, KDELR could bind CDNF and MANF via the C-terminal KDEL-like sequences (136, 140). The presence of KDEL-like sequence was required for the cardioprotective activity of recombinant CDNF in a rat model of myocardial ischemia (140). However, recombinant MANF lacking the RTDL sequence was neuroprotective in a rat model of stroke *in vivo* (133), suggesting that KDELR binding is not essential for the cytoprotective activity of extracellularly delivered MANF.

## CDNF and MANF molecular interactions

In addition to extracellular neurotrophic activities, CDNF and MANF are intracellular ER stress-regulated proteins that function in the ER. MANF promoter contains ER stress response elements (ERSE) I and ERSE II, and UPR transcription factors XBP1s and ATF6 can induce MANF expression (134, 141, 142). An ER stressor tunicamycin increased CDNF expression in cardiomyocytes *in vitro* (140) and in mouse kidney and liver *in vivo* (20), indicating that CDNF expression is also regulated by ER stress.

**Figure 4:**
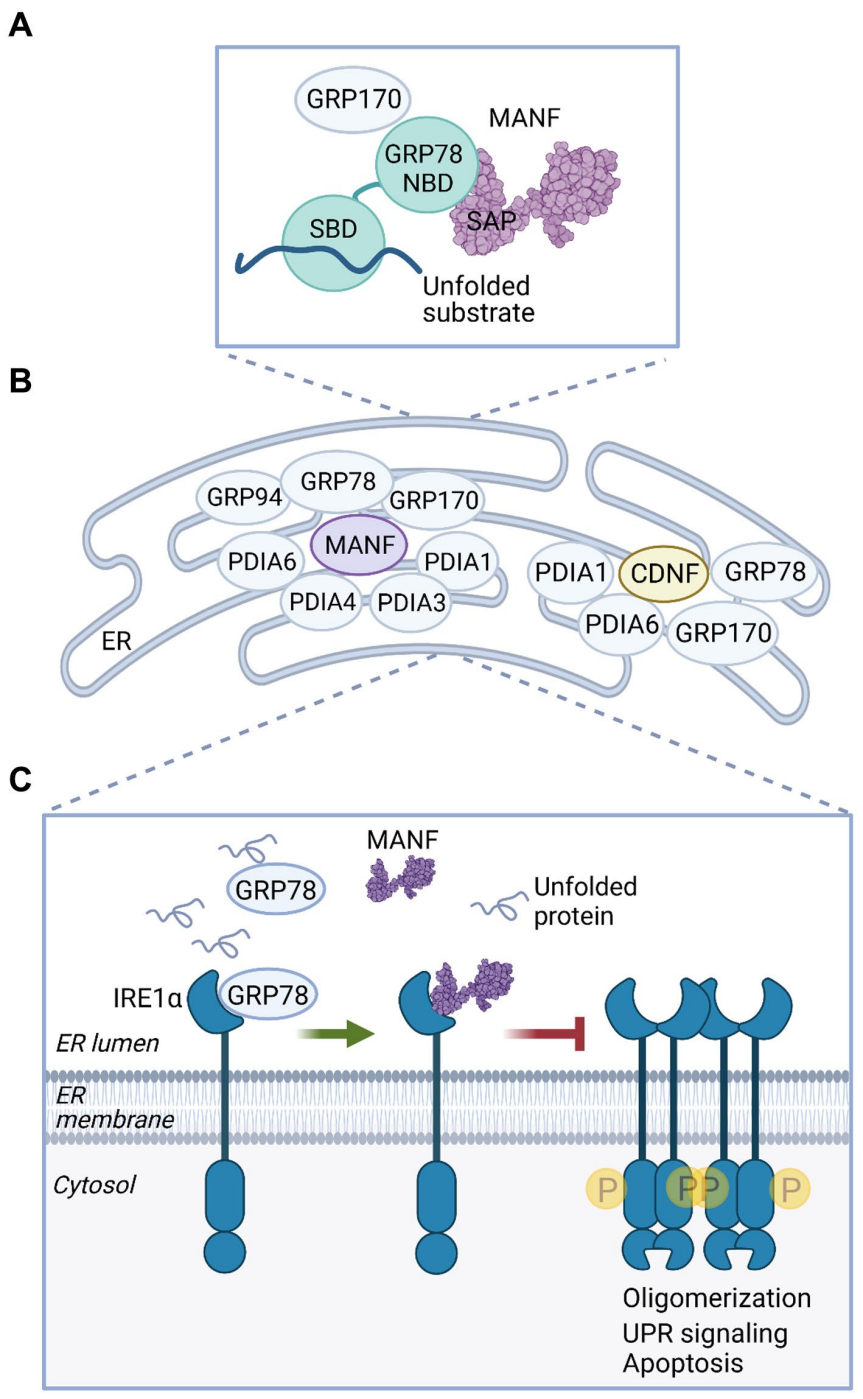
MANF and CDNF activities in the ER. **(A)** MANF functions as a nucleotide exchange inhibitor for GRP78. MANF (PDB ID: 2KVD) (126) interacts with the nucleotide-binding domain (NBD) of GRP78 via the C-terminal SAP-like domain and prolongs GRP78 interaction with a substrate protein, thus promoting protein folding homeostasis in the ER (144). GRP170 is a nucleotide exchange factor of GRP78 and was identified as an interaction partner of MANF and CDNF (20, 87, 145). SBD, substrate binding domain. **(B)** MANF and CDNF are members of the ER-localized multiprotein complex of chaperones, as demonstrated in affinity-purification mass spectrometry experiments (20, 145). **(C)** MANF regulates IRE1α signaling under ER stress. When GRP78 is dissociated from IRE1α to bind unfolded proteins, MANF is able to bind IRE1α. MANF binding inhibits IRE1α phosphorylation, oligomerization, and downstream signaling, preventing cell death (119).

Mechanistic studies on CDNF/MANF activity in ER stress and UPR indicate that an important functional partner for MANF, and also for CDNF, is the ER chaperone GRP78. Protein–protein interaction studies have demonstrated that both MANF (143–145) and CDNF (20) bind GRP78. In the first study, calcium-dependent interaction of MANF with GRP78 was shown to regulate MANF secretion (143). Later, it was suggested that MANF functions as a nucleotide exchange inhibitor (NEI) of GRP78 in the ER (Figure 4A). MANF could prolong GRP78 interaction with its client proteins, which would help to maintain protein folding homeostasis in the ER (144). Interaction of MANF and GRP78 was localized between the C-terminal domain of MANF and the nucleotide-binding domain (NBD) of GRP78, indicating that MANF is indeed a cofactor and not a client of GRP78 (144, 145). Interestingly, MANF can bind ATP, and the presence of ATP prevents the interaction of MANF with GRP78, as tested with purified proteins (145). However, the functional importance of the MANF-ATP interaction is not clear and warrants further studies. Differently from what one might expect, interaction with GRP78 was not needed for the neuroprotective activity of MANF. MANF mutants unable to bind GRP78 (144) still promoted the survival of dopamine and SCG neurons under ER stress *in vitro* (119, 145), and a MANF mutant unable to protect neurons perfectly bound GRP78 (119).

MANF and CDNF protein–protein interactomes were characterized using affinity purification-mass spectrometry (AP-MS) in human embryonic kidney HEK293 and rat insulinoma INS1 cell lines induced to overexpress MANF or CDNF (20, 145). From both HEK293 and INS1 cell lines, GRP78, GRP170, PDIA1, and PDIA6 were identified as ER-localized interaction partners for MANF (Figure 4B), and MANF interaction with GRP78, GRP170, and PDIA6 in the ER was also verified by bimolecular fluorescence complementation assay (BiFC) in cells (145). This finding was in accordance with other studies reporting GRP170 and PDIA6 as MANF interacting proteins (87, 144). GRP170 functions as a nucleotide exchange factor for GRP78 (146), and it is possible that MANF interaction with GRP170 is mediated by GRP78. PDIA1 and PDIA6 are members of the large family of protein disulfide isomerases (PDIs) that catalyze oxidative protein folding in the ER, a process where disulfide bonds are formed and proteins acquire native conformation (147). In addition to the abovementioned partners, GRP94, PDIA3, and PDIA4 were identified as potential MANF interacting chaperones and isomerases in HEK293 cells (145) (Figure 4B). These data suggest that MANF is a component of an ER-localized multiprotein complex containing several chaperones (145). Although MANF apparently does not have oxidoreductase activity (133), it may still have chaperone activity. MANF was shown to function as a chaperone specifically in reductive stress *in vitro*, and it was suggested that the cysteine residues of MANF are important for the chaperone activity (148).

Resembling results from MANF interactome analysis (145), characterization of ER-localized interactome of CDNF based on AP-MS analysis of HEK293 and INS1 cells identified GRP78, GRP170, PDIA1, and PDIA6 as potential ER luminal binding partners for CDNF (20) (Figure 4B). CDNF interaction with GRP78 and GRP170 in the ER was also verified using BiFC *in vitro*. Further analysis suggested that the interaction between CDNF and GRP78 takes place between the C-terminal domain of CDNF and the NBD of GRP78, which indicates that CDNF may function as a cofactor of GRP78 (20). However, the exact interaction interface between CDNF and GRP78 proteins remains to be defined. Based on these studies CDNF and MANF have common interaction partners in the ER, suggesting that they share similar activity mechanisms in cells.

A recent mechanistic study proposed a novel function for MANF as a direct regulator of UPR receptor IRE1α activation during ER stress (119) (Figure 4C). MANF was shown to directly bind to the luminal domain of IRE1α, suggesting that IRE1α functions as an ER-located receptor of MANF. MANF was shown to decrease ER stress-induced IRE1α phosphorylation, oligomerization, and downstream signaling. In cultured primary dopamine neurons, extracellularly added MANF decreased levels of *sXbp1* and *Atf6* under thapsigargin-induced ER stress. Importantly, the results suggested that MANF ability to interact with IRE1α was necessary for the neuroprotective activity of both extracellularly delivered MANF and intracellular, microinjected MANF during ER stress. Unlike WT MANF, mutant MANF unable to bind IRE1α neither promoted the survival of cultured primary dopamine neurons under thapsigargin-induced stress when added to the media nor promoted the survival of SCG neurons under tunicamycin-induced ER stress when MANF was microinjected into the cytoplasm. Interestingly, GRP78 prevented MANF binding to IRE1α, indicating that MANF and GRP78 binding sites may overlap in IRE1α. Thus, the authors suggested that MANF binds IRE1α under ER stress when GRP78 is dissociated from IRE1α and regulates IRE1α signaling to prevent irreversible ER stress, thus promoting cell survival. In addition to IRE1α, MANF also binds luminal domains of PERK and ATF6, albeit with lower affinity (119). However, the functional role of these interactions remains to be elucidated. Whether CDNF can interact with the UPR sensors similarly to MANF is unknown.

## Neuroprotection of CDNF and MANF by regulating UPR

Unlike classical NTFs, survival-promoting effects of exogenous CDNF and MANF on naïve dopamine neurons and other types of naïve neurons seem to be very low or possibly even non-existent (12, 20, 145). When CDNF was added to the culture media, it did not promote the survival of naïve primary SCG neurons, motoneurons, or DRG neurons *in vitro*, differently from the respective control NTFs, i.e., nerve growth factor, ciliary neurotrophic factor, and GDNF (12). However, under ER stress, CDNF and MANF show neuroprotective effects, suggesting that stress conditions are required for their activity. CDNF and MANF added to the culture media of mouse embryonic dopamine neurons increased neuron survival under thapsigargin-induced ER stress (20, 145). Furthermore, CDNF and MANF treatment significantly decreased the expression of UPR genes *sXBP1, Atf6* and *Grp78*, implying that CDNF and MANF can both regulate IRE1α and ATF6 UPR branches in primary dopamine neurons under ER stress (20, 145). Additionally, protective effects of recombinant MANF against ER stress-induced apoptosis were demonstrated in tunicamycin-treated primary cortical neurons in culture (149).

Intracellular CDNF or MANF, delivered by microinjection of recombinant protein or plasmid cDNA, promoted survival of SCG neurons under tunicamycin-induced ER stress (20, 145). Similarly, microinjection of MANF encoding cDNA plasmid promoted survival of DRG neurons under thapsigargin-induced ER stress (133). The survival-promoting effects of CDNF and MANF in SCG neurons were dependent on the activity of the PERK and IRE1α pathways (20, 145). These *in vitro* studies suggest that CDNF and MANF can promote neuron survival both as extracellular paracrine factors and as intracellular proteins, and both the extracellular and intracellular activities may engage UPR pathways. The survival-promoting activity of CDNF and MANF is possibly related to IRE1α and ATF6 pathways in dopamine neurons and IRE1α and PERK pathways in SCG neurons, suggesting that CDNF and MANF may regulate multiple UPR pathways in parallel (20, 145), and the regulatory activity may differ between different types of neurons and conditions of ER stress.

Alleviation of ER stress has been proposed to account for neuroprotective activity of CDNF and MANF also in animal models of PD. This possible mechanism was investigated in the study of Voutilainen and others (107) in a rat 6-OHDA model of PD, where GRP78 expression and phosphorylation of eIF2α were analyzed at 4 weeks post lesion and 4 h post-CDNF delivery. The results indicated that CDNF had a trend for decreasing both GRP78 expression and p-eIF2α levels relative to vehicle-treated rats (107). In a rodent model of intracerebral hemorrhage, by contrast, CDNF administration was shown to efficiently reduce expression of GRP78, ATF6α, ATF4, and CHOP in the hemorrhage area of the striatum (94). Furthermore, a recent study demonstrated that CDNF administration attenuated activation of all three UPR pathways in motoneurons isolated from the superoxide dismutase (SOD)-G93A mice model of amyotrophic lateral sclerosis (ALS) (150). Thus, CDNF is capable of downregulating UPR signaling *in vivo*. The involvement of ER stress in MANF-mediated neuroprotection of dopamine neurons has been investigated in *C. elegans*. The neuroprotective effect of MANF was lost when UPR genes were blocked by RNAi in the mutant αSyn overexpression model in nematodes, suggesting that MANF protects neurons via affecting UPR signaling (151).

Striatal administration of MANF was shown to increase SOD activity and glutathione production in the MPTP model of PD, demonstrating antioxidative activity of MANF (122). MANF was also shown to increase levels of antioxidant regulator NRF2 and promote the translocation of NRF2 to the nucleus (152). Moreover, MANF increased activation of the AKT/GSK3β-Nrf-2 signaling axis in the 6-OHDA model of PD *in vivo*, suggesting that this would serve as a potential neuroprotective mechanism (121). Furthermore, a recent study revealed that CDNF could also increase transcriptional activity of NRF2 in an experimental model of intracerebral hemorrhage (94).

## MANF and CDNF in inflammation

Microglia is a major immune cell type that functions in inflammatory response in the CNS. Resident microglia are constantly monitoring the tissue environment in the brain parenchyma in search of damage. In response to injury, microglia are activated and acquire macrophage morphology. Activated microglia release various factors including pro-inflammatory and anti-inflammatory cytokines, produce reactive oxygen and nitrogen species, and function in phagocytosis (153). Inflammatory cytokines produced by activated microglia participate in the progression of PD pathogenesis (154).

Increasing evidence suggests that CDNF and MANF possess immunomodulatory functions that contribute to their cytoprotective and neuroprotective properties (56, 88, 94, 111, 155). Although MANF was originally isolated as a secreted factor from the culture medium of a mesencephalic astrocyte cell line (18), it was noted that in normal conditions MANF expression is low in brain glial cells but can be induced upon glial cell activation in stress conditions (156). *In vitro*, an increase in MANF expression was shown in cultured primary microglia, astrocytes, and oligodendrocytes upon treatment with ER stressor tunicamycin (156).

Evidence indicates that MANF can attenuate inflammatory response by repressing NF-κB signaling in ER stress and inflammatory conditions (71, 157, 158). A recent study identified neuroplastin (NPTN) as a receptor of MANF on the cell surface (159). In the presence of NPTN, MANF was shown to repress ER stress-induced NF-kB activation and expression of pro-inflammatory *IL-6* and *Cxcl-1*, and pro-apoptotic *Chop in vitro*. The authors suggested that MANF binding to NPTN reduces inflammation, ER stress, and subsequent cell death (159). It was also suggested that MANF could directly regulate NF-κB signaling in cells. MANF may enter the cell nucleus upon ER stress and interact via the C-terminal SAP domain with the DNA binding domain of p65 subunit of NF-κB, and thus, prevent transcription of NF-κB target genes (71). In a subsequent study, the authors suggested that SUMO1 promotes nuclear import of MANF and regulates MANF interaction with p65 (158).

Unlike MANF, CDNF does not bind to NPTN (159) but shows anti-inflammatory effects. Lentivirus vector-mediated overexpression of CDNF in cultured primary astrocytes was demonstrated to decrease ER stressor tunicamycin-induced astrocyte damage and secretion of inflammatory cytokines IL-1β, IL-6, and TNFα (160). CDNF treatment decreased secretion of pro-inflammatory prostaglandin E2 and IL-1β from LPS-induced primary microglia and prevented LPS-induced inflammatory injury in the microglia by inhibiting phosphorylation of JNK (161). CDNF treatment was also reported to decrease inflammatory response in LPS-induced primary microglia via inhibiting Akt phosphorylation and FoxO1/mTOR signaling (162). In a study by Nadella and others (155), the effect of CDNF on inflammation levels was investigated *in vivo* in the 6-OHDA model of PD. CDNF overexpression via a plasmid vector reduced activated microglia and IL-6 levels in the SN. As mentioned before, CDNF co-administration was shown to attenuate neuroinflammation and promote survival of dopamine neurons in grafted fetal ventral mesencephalic tissue that was transplanted into the striatum of 6-OHDA-treated hemiparkinsonian rats (111). In addition, CDNF administration decreased recruitment of microglia/macrophages to the graft and decreased production of pro-inflammatory cytokine IL-1β and TNFα levels in the grafted striatum. *In vitro* studies using 6-OHDA-stimulated BV2 microglial cell line indicated that CDNF treatment decreased pro-inflammatory markers iNOS, CD11b, and IL-6, while increasing expression of IL-10 and Arg-1, suggesting that CDNF treatment enhances microglial polarization toward pro-repair state macrophages (111). The ability of CDNF to attenuate pro-inflammatory cytokines was also demonstrated in the intracerebral hemorrhage model, where administration of CDNF reduced levels of IL-6, TNF-α, IL-1β, and IFN-γ, while increasing the level of IL-10 in the peri-hematoma striatum on days 1 and 3 after hematoma induction (94). Similarly, intracerebroventricular delivery of CDNF after ischemic stroke induction in mice decreased production of TNF-α and IL-1β (163). Thus, CDNF may have immune modulatory effects in the CNS, but the exact mechanisms need to be identified.

## Discussion

The biology of endogenous CDNF and MANF proteins in the mammalian dopamine system has remained largely unknown until recent years when papers describing the effects of CDNF and MANF depletion in the mouse dopaminergic system on the CNS and ENS were published (52, 81, 85, 90).

Collectively, the results from different animal models show that the role of endogenous CDNF and MANF in the brain and specifically in the dopaminergic system varies between model organisms. In mice, loss of endogenous CDNF, MANF, or both does not impair the survival of the nigrostriatal dopamine neurons (52, 81, 90). However, loss of mouse CDNF does lead to a functional defect in the nigral dopamine neurons and to specific degeneration of enteric dopamine neurons (81, 85). Differently from *Manf*^−/−^ mice, in the fruit fly and zebrafish, MANF deletion leads to defects in development of dopamine neurons (95, 102). Dopaminergic phenotype was also detected in zebrafish lacking CDNF (103). These differences in observed dopaminergic phenotypes can be partially explained by different tissue expression patterns of CDNF and MANF in the model organisms. In the developing fruit fly, DmMANF is expressed by glial cells (95), while in the two-week-old mouse brain, MANF is already highly expressed in neurons (51).

The main brain phenotype of *Cdnf^−/−^* mice associates CDNF with DAT activity (81). Amphetamine-induced hyperactivity response of *Cdnf^−/−^* mice is notable and *ex vivo* experiments explain them partially. However, understanding of the mechanism for how CDNF regulates the reverse activity of DAT is lacking. Whether CDNF induces post-translational modifications in DAT in response to amphetamine, altering its activity or plasma membrane localization, is not known. Moreover, the effect of CDNF on dopamine synapses should be studied in detail.

The indispensability of CDNF in the development and maintenance of enteric dopamine neurons vs. the dispensability of CDNF in the maintenance of midbrain dopamine neurons in mice is a paradox warranting further investigations. Midbrain dopamine neurons originate from the mesodiencephalic floor plate and are formed earlier during development than the enteric dopamine neurons that originate from the neural crest in mammals (4). However, these neurons share a similar dopamine-related gene expression pattern and requirement of similar factors, such as GDNF, TGF-β and BMP, for development, function, or survival in the long term (4). Comparison of transcriptomes between nigral and enteric dopamine neurons of *Cdnf^−/−^* mice has not been performed but could assist in the analysis of increased vulnerability of enteric dopamine neurons lacking CDNF. It is important to note that CDNF is expressed in most enteric dopamine neurons (85), while no studies have identified co-localization of CDNF in nigral dopamine neurons. There are no reports about alterations in the number of enteric dopamine neurons of PD patients (4). Furthermore, expression of CDNF in enteric neurons of PD patients has not been evaluated; however, colonic biopsies could be informative regarding potential changes in CDNF levels. Since loss of CDNF impairs gut functionality (81, 85), one might ask whether CDNF administration would improve functionality and alleviate constipation in PD patients.

It would be interesting to ascertain the degree of similarity/difference in the biological roles of CDNF and MANF. CDNF is present only in vertebrates, while MANF is highly evolutionarily conserved and described even in sponges belonging to the phylum *Porifera* (164). The phenotypes of *Cdnf^−/−^* and *Manf^−/−^* mice are strikingly different (25, 81), and the phenotype of *Cdnf^−/−^*::*Manf^−/−^* mice resembles *Manf^−/−^* mice, indicating no significant redundancy between CDNF and MANF (52). In the skeletal muscle, there seems to be functional compensation between CDNF and MANF since *Cdnf^−/−^::Manf^−/−^* mice have exacerbated UPR activation compared with *Cdnf^−/−^* and *Manf^−/−^* mice (52). While Cordero-Llana and others (109) suggested a synergistic neurorestorative effect of virally delivered CDNF and MANF on nigral dopamine neurons in a 6-OHDA model of PD, simultaneous deletion of endogenous CDNF and MANF did not result in loss of nigral dopamine neurons in aged mice (52). To further elucidate roles of CDNF and MANF in the midbrain dopamine system, studies using CDNF- and MANF-ablated mice in PD models could be informative. Possibly, loss of CDNF, MANF, or both can increase the vulnerability of midbrain dopamine neurons to toxin-induced stress in PD models similarly to that demonstrated using ER stressors to neurons lacking MANF (90). Thus far, the role of CDNF and MANF in the maintenance of midbrain dopamine neurons in rodents has been studied using models of embryonic gene deletion, which might result in compensatory mechanisms during mouse development, masking the direct effects of the gene deletion. Therefore, it would be interesting to generate mouse models where CDNF and MANF are ablated in adult mice.

Based on studies using human post-mortem brain tissue, UPR activation seems to be linked to pathophysiology of PD. However, it is not known whether changes in endogenous MANF or CDNF expression in the brain tissue are associated with UPR activation in PD patients. Therefore, it would be informative to assess MANF and CDNF expression in human brain tissue in relation to αSyn pathology and UPR markers. An interesting question is whether MANF and CDNF could be used as biomarkers for PD since both proteins can be detected in blood circulation, which is an easily accessible matrix. Increased levels of circulating MANF were detected in PD (54) and acute ischemic stroke (61), conditions related to the brain. When ER stress-regulated proteins in the blood were studied as potential biomarkers for PD, it was found that MANF together with three other proteins, PDIA1, PDIA3, and clusterin in combination with age and gender confounders were able to discriminate PD patients from the non-PD group (165). These studies suggest that the biomarker potential of MANF in PD should be investigated further. An open question is, of course, whether MANF levels in the blood can directly reflect brain physiology or is the supposed association of MANF with PD pathophysiology indirect. It might be useful to investigate MANF and CDNF levels in cerebrospinal fluid, which should better reflect biochemical changes in the brain of PD patients.

Resolving the mechanism of CDNF and MANF neuroprotective action is important for the development of efficient CDNF/MANF-based therapies for PD. CDNF and MANF can function both inside the cells in the ER and extracellularly, possibly in a paracrine or autocrine manner *in vivo*. Both modes of action, extracellular and intracellular, can alleviate ER stress in neurons. Furthermore, *in vitro* studies in neurons suggest that MANF and CDNF can decrease the activity of two or even three UPR pathways in parallel to promote neuron survival under ER stress (20, 119, 145). Characterization of molecular mechanism of CDNF/MANF in UPR regulation and its timing in relation to acute or chronic ER stress events would be informative and could help to develop targeted MANF/CDNF-based interventions to modulate activity of UPR pathways and enhance neuronal survival, possibly also in PD patients in the future.

After many years of speculation about possible lipid binding (166), MANF interaction with sulfatide lipid was reported in 2018 (98). However, elucidation of the role of sulfatide binding in MANF uptake and cytoprotective activity against ER stress necessitates further investigations. Mechanistic information about how MANF-sulfatide is internalized to cells, where it is localized in the cells, and how it alleviates ER stress is currently lacking. CDNF ability to bind lipids is completely unknown. Moreover, NPTN, the novel plasma membrane receptor for MANF, does not bind CDNF (159), suggesting that CDNF has other receptors on the cell surface. Structural features that determine MANF binding to NPTN have not yet been fully characterized.

In the ER lumen, two important mechanistic roles for MANF were recently reported. Firstly, it was suggested that MANF interacts with GRP78 as a collaborator that functions as a NEI of GRP78, thus helping to maintain protein homeostasis (144). Secondly, MANF was shown to directly interact with IRE1α and decrease IRE1α activity in chronic ER stress to promote cell survival (119). Since the MANF binding site to IRE1α overlapped with that of GRP78, and GRP78 binding to IRE1α was stronger than that of MANF, the authors suggested that MANF could bind to IRE1α only when GRP78 is dissociated, as is the case under severe ER stress (119). An additional feature of MANF is that it can interact either directly or indirectly with a set of chaperones and isomerases in the ER (145), being involved in regulation of protein folding homeostasis. How these additional interactions affect the above-mentioned functions of MANF remains unknown. For example, MANF partners PDIA1 and PDIA6 also regulate IRE1α activity (167, 168). The potential chaperone activity of MANF itself (148) also requires further investigations.

Increasing evidence indicates that the cytoprotective activities of MANF and CDNF are related to their ability to dampen inflammatory response in microglia and astrocytes by reducing expression and secretion of proinflammatory cytokines. MANF was reported to regulate activity of the NF-κB pathway (71, 157, 158), and NPTN was shown to mediate the anti-inflammatory effect of MANF (159). Immune modulatory effects of MANF on macrophage polarization toward pro-repair phenotype in tissue injury have been described (88), and CDNF may also affect polarization of microglia/macrophages *in vitro* (111). Aging is related to impairment in the regulation of immune system function, leading to chronic, non-resolved low-grade inflammation. Dysregulated immune response can contribute to the gradual development of PD years before the start of clinical motor symptoms (154). Moreover, alterations in MANF levels have been reported in autoimmune and inflammatory diseases (53, 67, 71) (Table 1). Investigations of the roles of CDNF and MANF in the immune system, inflammatory response, and in association with UPR would be useful for developing future therapies also for PD.

When evaluating the therapeutic potential of CDNF in PD, it is important to consider possible side effects. CDNF and MANF delivered to brain parenchyma do not have any obvious impact on naïve rodent brains, suggesting a low risk for therapeutic side effects. Interestingly, CDNF has been shown to improve long-term memory in both WT mice and APP/PS1 mice, modeling Alzheimer’s disease (169). The mechanism behind improved memory was not identified, but no association emerged between CDNF and neurogenesis in the hippocampus or amyloid pathology. Recently, CDNF administration was demonstrated to increase *Bdnf* levels in the hippocampus in a N171-82Q mouse model of Huntington’s disease (170). Thus, the effects of CDNF on memory and cognitive functions as well as on BDNF signaling certainly warrant further investigations.

Recently, MANF was associated with rejuvenating effects in chronic parabiosis experiments in mice, suggesting that MANF has anti-aging properties (56). Thus, one could consider how to increase endogenous levels of CDNF and MANF in humans to promote healthy aging and possibly prevent or delay the development of neurodegenerative diseases. Dietary restriction and exercise are potential ways to increase health span (171, 172). Circulating MANF levels were shown to increase in therapeutic fasting in humans, suggesting that MANF is associated with fasting-induced beneficial effects (55). Interestingly, exercise was reported to induce endogenous CDNF expression in the cerebral cortex of naïve rats and in the striatum of 6-OHDA-lesioned rats (173, 174). Exercise was also shown to increase CDNF expression in the rat spinal cord in a 6-OHDA model of PD (175), indicating that exercise might have a broader effect on CDNF regulation in the CNS. These findings highlight the potential of exercise as a non-pharmacological approach to increase CDNF expression.

In conclusion, CDNF and MANF are neuroprotective proteins that can modulate UPR signaling and inhibit inflammatory processes, both of which are potentially relevant to PD pathogenesis. Since ER stress and inflammation are interconnected processes, deeper understanding of their complex interplay and the potential roles of CDNF and MANF in these processes, could benefit development of new therapeutic strategies for PD.

## Author contributions

PL drafted the manuscript. EP and PL wrote the paper, prepared figures, edited the manuscript, and accepted the final version. All authors contributed to the article and approved the submitted version.

## Funding

This work was financially supported by the Academy of Finland (grant 343299), the Jane and Aatos Erkko Foundation, and Cure Parkinson’s Trust UK. Open access funding was provided by University of Helsinki Library.

## Conflict of interest

PL is an inventor in the CDNF- and MANF-related patents (7,452,969; 9,592,270) owned by Herantis Pharma Plc.

The remaining author declares that the research was conducted in the absence of any commercial or financial relationships that could be construed as a potential conflict of interest.

## Publisher’s note

All claims expressed in this article are solely those of the authors and do not necessarily represent those of their affiliated organizations, or those of the publisher, the editors and the reviewers. Any product that may be evaluated in this article, or claim that may be made by its manufacturer, is not guaranteed or endorsed by the publisher.
